# Supplementary material for: The Effect of Phenyl Content on the Liquid Crystal-Based Organosilicone Elastomers with Mechanical Adaptability
Source: Polymers (Basel). 2022 Feb 24;14(5):903. doi: 10.3390/polym14050903 (PMC8912632; doi:10.3390/polym14050903)
Supplement: Supplementary file 1 [file polymers-14-00903-s001.zip › polymers-1528999-supplementary.pdf]

polymers

MDPI

Supplementary Materials

The Effect of Phenyl Content on the Liquid Crystal-Based Organosilicone Elastomers with Mechanical Adaptability

Zhe Liu, Hua Wang and Chuanjian Zhou *

School of Materials Science and Engineering, Shandong University, Jinan 250061, China.
201820362@mail.sdu.edu.cn (Z.L.); hwang@sdu.edu.cn (H.W.)
* Correspondence: zhouchuanjian@sdu.edu.cn

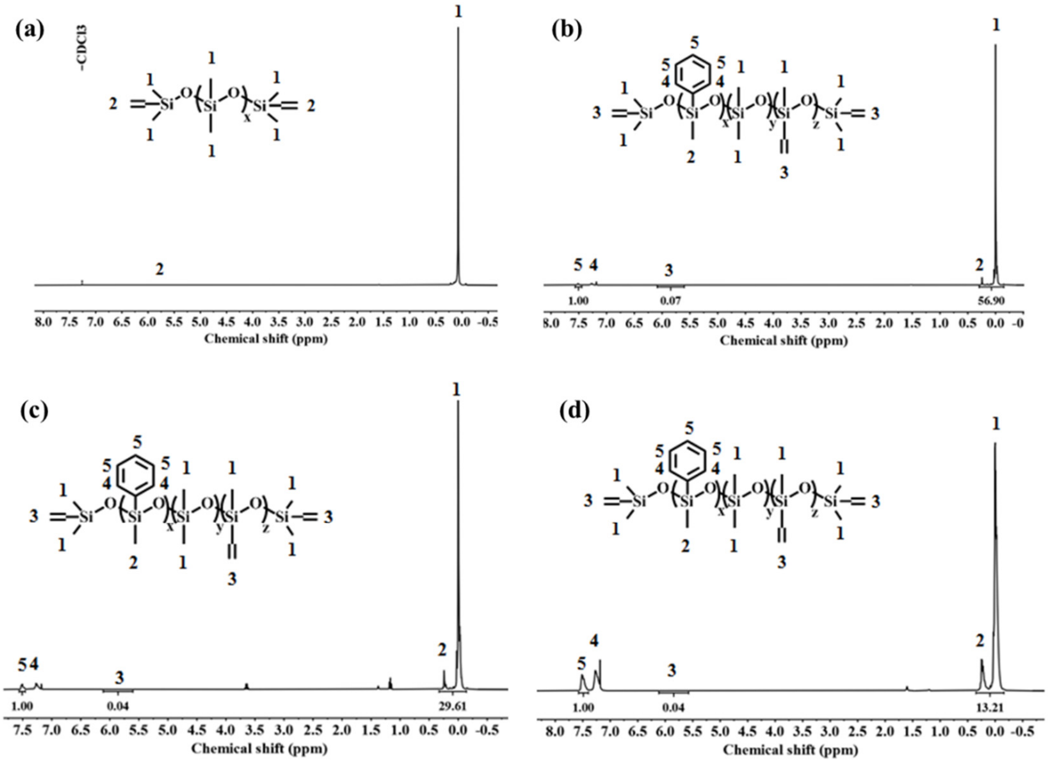

Figure S1. ^{1}H NMR spectra of (**a**) Silicone oil with 0% phenyl content; (**b**) Silicone oil with 5% phenyl content; (**c**) Silicone oil with 10% phenyl content ; (**d**) Silicone oil with 20% phenyl content.
